# Childhood disability population-based surveillance: Assessment of the Ages and Stages Questionnaire Third Edition and Washington Group on Disability Statistics/UNICEF module on child functioning in a rural setting in South Africa

**DOI:** 10.4102/ajod.v5i1.265

**Published:** 2016-09-26

**Authors:** Marieta Visser, Mariette Nel, Caretha Bronkhorst, Lara Brown, Zaskia Ezendam, Kira Mackenzie, Deidré van der Merwe, Marné Venter

**Affiliations:** 1 Department of Occupational Therapy, Faculty of Health Sciences, University of the Free State, Bloemfontein, South Africa; 2 Department of Biostatistics, Faculty of Health Sciences, University of the Free State, Bloemfontein, South Africa

## Abstract

**Background:**

Epidemiological information on childhood disability provides the basis for a country to plan, implement and manage the provision of health, educational and social services for these vulnerable children. There is, however, currently no population-based surveillance instrument that is compatible with the International Classification of Functioning, Disability and Health (ICF), internationally comparable, methodologically sound and comprehensively researched, to identify children under 5 years of age who are living with disability in South Africa and internationally. We conducted a descriptive pilot study to investigate the sensitivity and specificity of translated versions of the Ages and Stages Questionnaire Third Edition (ASQ-III) and the Washington Group on Disability Statistics/UNICEF module on child functioning (WG/UNICEF module) as parent-reported measures. The aim of our study was to identify early childhood disabilities in children aged 24–48 months in a rural area of South Africa, to determine the appropriateness of these instruments for population-based surveillance in similar contexts internationally.

**Methods:**

This study was conducted in the Xhariep District of the Free State Province in central South Africa, with 50 carers whose children were registered on the South African Social Security Agency (SASSA) database as recipients of a grant for one of the following: Care Dependency, Child Support or Foster Care. The researchers, assisted by community healthcare workers and SASSA staff members, conducted structured interviews using forward–backward translated versions of the ASQ-III and the WG/UNICEF module.

**Results:**

Both measurement instruments had a clinically meaningful sensitivity of 60.0%, high specificity of 95.6% for the ASQ-III and 84.4% for the WG/UNICEF module, and the two instruments agreed moderately (Kappa = 0.6).

**Conclusion:**

Since the WG/UNICEF module is quicker to administer, easier to understand and based on the ICF, it can be considered as an appropriate parent-reported measure for large-scale, population-based as well as smaller, community-specific contexts. It is, however, recommended that future research and development continues with the WG/UNICEF module to enhance its conceptual equivalence for larger-scale, population-based studies in South Africa and internationally.

## Introduction

Early childhood development plays a vital role in health and social outcomes and forms the basis of human capital (Grantham-McGregor, Cheung, Cueto, Glewwe, Richter, Strupp. 2007; Heckman & Mosterov, 2007; Wadsworth & Butterworth 2005). Internationally, there is a call for professionals, parents and policy makers to renew their focus on early childhood development (Britto, Yoshikawa & Boller 2011; Irwin, Siddiq & Hertzman 2007; Regalado & Halfon 2001; Richter *et al*. 2014; South African Human Rights Commission/UNICEF 2011).

Developmental delays and disabilities prevent millions of children from achieving their optimal potential (Grantham-McGregor *et al*. 2007; Walker *et al*. 2007). In 2004, 93 million children (0.5% of the children in the world), 14 years of age or younger, lived with a moderate or severe disability of some kind (UNICEF 2013). More recently, it has been found that internationally, more than 200 million surviving children have developmental delays or disabilities (Scherzer *et al*. 2012). Statistically sound epidemiological information is imperative in order to implement and manage the provision of health, educational and social development services for these children (Hack *et al*. 2005).

However, noticeable discrepancies between countries’ data sets, different and often non-comparable survey methods and insufficient epidemiological information on childhood disability represent three problems that are of global concern (Elwan 1999; UNICEF 2013; WHO 2014). Moreover, in developing nations such as South Africa, evidence regarding children with disabilities is often unreliable and unavailable (African Child Policy Forum [ACPF] 2011; UNICEF 2012; WHO 2002).

Reasons for this lack of reliable childhood disability statistics include, firstly, that there are obvious discrepancies between the results obtained from the questions and methods used in population-based surveys such as the National Census (Statistics South Africa 2003), General Household Surveys (Statistics South Africa 2010, 2013), Community Surveys, Multiple Indicator Cluster Surveys (MICS) (UNICEF 2012), Global Burden of Diseases (GBD) studies and World Health Surveys (WHS), to name a few (Elwan 1999; Health Systems Trust 2011; UNICEF 2013). Secondly, numerous countries focus on evidence regarding adults, as clearly observed in surveys such as the GBD and WHS (including in the 2011 South African National Census). Consequently, children younger than 5 years have been excluded, resulting in insufficient current statistical data regarding the prevalence of early childhood disabilities internationally (Statistics South Africa 2014a).

Possible causes for the threefold problem mentioned above may include (1) the complexity of the term ‘disability’ (Molden & Tøssebro 2012), (2) the difficulty in detecting disabilities of children under 5 years (Molden & Tøssebro 2012) and (3) the lack of a population-based surveillance instrument that is compatible with the International Classification of Functioning, Disability and Health (ICF) (WHO 2007), internationally comparable, methodologically sound and comprehensively researched, to identify disabled children under 5 years of age living in South Africa and internationally (Elwan 1999; Madans, Loeb & Altman 2011).

The consequence of not having the appropriate surveillance measures required to detect childhood disability (for smaller, population-based studies or large census surveillance) is that no internationally comparable epidemiological information can be obtained. Such epidemiological data form the basis for countries to plan, implement and manage their provision of health, educational and social services, inform appropriate guiding policy initiatives and address social justice and human rights issues for these vulnerable children (Hack *et al*. 2005).

In order to comply with global guiding legislative and policy documents, various initiatives have been undertaken by organisations such as WHO, UNICEF and Washington Group on Disability Statistics (WG) (WHO 2011). As a result, an appropriate measurement instrument, based on the ICF (adopted as the conceptual framework for the World Report on Disability (WHO & World Bank 2011) and embodied in the United Nations Convention of Rights of Persons with Disabilities) and called the Washington Group on Disability Statistics/UNICEF module on child functioning (WG/UNICEF module), has been developed (WG/UNICEF 2014). However, this instrument, developed to be internationally comparable and methodologically sound, had not (at the time of the research presented here) been fully vetted and finalised that would allow its suitability for various global contexts, for example, in South Africa (WG/UNICEF 2014).

As a member state of the United Nations (UN), South Africa is held accountable for the provision of epidemiologically sound information regarding childhood disability in order to give effect to the Convention on the Rights of Persons with Disabilities (WHO 2011). Although no population-based measurement question and/or measure to detect childhood disabilities is currently available, Stats SA remodelled its approach to align their General Household Survey and National Census 2011 questionnaires with the WG/UNICEF module to measure childhood disability (Madans *et al*. 2011; Statistics South Africa 2014b). The outcome of this remodelling process could ensure future inclusion of previously excluded children under 5 years, in order to provide sound epidemiological information (Department of Social Development/Department of Woman Children and People with Disabilities Republic of South Africa/UNICEF 2012).

In order to manage childhood disabilities in South Africa, there is a call for leadership from researchers, practitioners, governments and international organisations to fulfil their responsibility to understand and measure childhood disability, enabling every child to achieve his/her optimal potential (Scherzer *et al*. 2012).

## Theoretical overview

### Childhood disability epidemiology

According to the 2001 South African Census, which included children under 5 years of age, 5% of the total population have some form of disability (ACPF 2011; Statistics South Africa 2003). Furthermore, 2.5% of children under 18 years and 1.6% of children under 4 years were reported to have a disability (ACPF 2011; UNICEF 2012).

A community survey conducted by Statistics South Africa (Stats SA) in 2007 indicated that 0.9% of children under 4 years have a disability (Health Systems Trust 2011). The General Household Surveys of 2008 and 2009 reported 0.6% and 11.2%, respectively, of the total child population as disabled. The prevalence of disability appeared unusually high for young children under 4 years (27.5%), and in the age group 5–9 years, 10% were classified as disabled (Statistics South Africa 2010, 2013; UNICEF 2012).

It is argued that these discrepancies in national data could be ascribed, firstly, to the variety in the questions of the population-based surveys used to determine disability (Molden & Tøssebro 2012). Secondly, the child mortality rate in South Africa of 47 per 1000 live births of children under 5 years – ‘the probability of dying by age five expressed as the total number of such deaths per 1000 live births’ (WHO 2013:22) – might falsely portray low childhood disability prevalence (Naledi, Barron & Schneider 2011). Thirdly, mild-to-moderate disabilities may be detected only once a child enters primary school or even as late as secondary school in some cases (Naledi *et al*. 2011).

Lastly, the complexity of the term ‘disability’ and the use of different types of wording to ask about disability have been identified as a primary reason for large discrepancies in epidemiological information on childhood disability between countries (Schneider 2009). Disability is a complex phenomenon which cannot be captured in a single definition. It is defined, according to the ICF, as ‘an outcome of the interaction of a health condition and the context of the person with the health condition’ (Schneider, Barron & Fonn 2007:273). The outcome of the interaction is described at three levels of functioning, namely the body, the person and society (Schneider *et al*. 2007; WHO 2001). This definition is in agreement with the biopsychosocial model of health, which suggests that one should look at both the individual and the environment when a person’s experience of disability is described accurately and comprehensively (Duncan *et al*. 2009).

Comparability of international and national epidemiological information could be improved, if the above definition and perspective are adopted and used in measurement instruments by all member countries of the UN. The implications of this for population-based surveys would be to incorporate questions covering all ICF levels of functioning. However, in the self-reported context, as is the case in censuses and general household surveys, it is important to select a level of functioning that a person can report on most accurately. This selection of a level of functioning has been identified as being the ‘person level’ of activity (Schneider *et al*. 2007; WHO 2001) as reflected in the census and surveys developed by the WA and South African researchers (Schneider *et al*. 2009).

In addition to epidemiological information, population-based surveys can also serve as an effective way to detect developmental delays and/or disabilities in young children. Research strongly suggests the use of surveys such as parent-reported screening instruments (Glascoe & Marks 2011). Parent-reported measures can also be applied as an early detection method in rural communities that do not have access to specialised treatment (Scherzer *et al*. 2012).

It has been recognised, however, that although population-based surveys can provide valuable epidemiological information, it remains important for a country to also incorporate early assessment and treatment into routine paediatric care programmes to ensure that basic health needs and human rights are met (Scherzer *et al*. 2012). If such paediatric care programmes had been in place in South Africa, the health sector’s over-investment in remediation of established disabilities at later ages and under-investment in health promotion through the early detection of disability could have been corrected (Heckman 2007).

## Measurement instruments

### Ages and Stages Questionnaire third edition

The earlier that intervention programmes are implemented for a child with disability, the better the potential benefit (Engle *et al*. 2007; Grantham-McGregor *et al*. 2007). Furthermore, early detection, support and treatment have positive effects on affective, cognitive, motor and social development (Scherzer *et al*. 2012).

There is clear evidence for the effectiveness of screening for a number of conditions, many of which are readily detectable in the neonatal period (Oberklaid, Wake, Harris, Hesketh & Wright. 2002). Developmental screening is described as a brief systemic process where delays are identified in children to determine whether further assessment is required (Rydz *et al*. 2006). Surveillance, however, is an ongoing, accumulative process enabling healthcare workers to detect early childhood developmental delays and/or disabilities (Committee on Children with Disabilities 2001).

Of the many available parent-reported measurement instruments, the Ages and Stages Questionnaire Third Edition (ASQ-III) and the WG/UNICEF module were selected for this study. Although there are no other known population-based, parent-reported survey instruments compatible with the ICF other than the WG/UNICEF module, instruments such as the Ten Question Screen (TQ), Ten Question Plus (TQP) and the Malawi Developmental Assessment Tool (MDAT) (Gladstone *et al*. 2010) have been used in several population-based studies (Durkin, Davidson, Desai, Hasan, Kahan, Shrout. 1994; Ibrahim & Bhutta 2013; Wu *et al*. 2010). The TQ has been used in MICS between 2000 and 2010 and identified as a rapid, low-cost screening instrument (UNICEF 2013). The TQ is sensitive to identify serious cognitive, motor and seizure disability, but not vision and hearing disability (Durkin *et al*. 1994; Maulik & Darmstadt 2007), and it requires follow-up medical and developmental assessment to confirm disability. More clinical developmental measurement instruments, often used to confirm disability identified in surveillance, include the Bayley Scales of Infant Development (Rademeyer 2010) and the Denver Developmental Screening Test (Gladstone *et al*. 2008).

The ASQ-III has been standardised on children aged from one month to five-and-a-half years in the USA (Squires *et al*. 2009). The ASQ-III is a clinical-based, parent-reported instrument to determine a child’s developmental milestones, and consequently, to identify developmental delays (Squires *et al*. 2009). The ASQ-III contains simple questions that require the primary carer to select the most appropriate response based on previous observations of the child’s ability or inability to perform daily activities expected of the specific age group (Tsai *et al*. 2006). The instrument consists of five domains, namely communication, gross- and fine motor, problem-solving and personal–social (Tsai *et al*. 2006). This screening and surveillance instrument is considered cost-effective and appropriate for developing countries with low socioeconomic climates (Gollenberg *et al*. 2009; Squires *et al*. 2009). It is, however, important to consider translation and development of conceptually equivalent versions for each specific population because some of the questions involve culture-specific concepts. It can be used to survey large numbers of children, but the administration thereof can prove time-consuming (Rydz *et al*. 2006).

Previous versions of the ASQ have been researched in other studies, occasionally in combination with other measures such as the TQ, Parents’ Evaluation of Developmental Status (PEDS) and the Bayley Scales of Infant Development II/BSID II (Gollenberg *et al*. 2009; Limbos & Joyce 2011). As reported by Gollenberg *et al*. (2009), the ASQ-III has a sensitivity of 75% and a specificity of 86%. However, no studies using the ASQ-III in a rural South African context have been documented yet.

## The Washington Group on disability statistics/UNICEF module on child functioning

The WG, in partnership with UNICEF and other stakeholders, has been working with the aim to improve the quality and international comparability of disability measures suitable for censuses and national surveys (Madans *et al*. 2011; Madans & Loeb 2013; WG/UNICEF 2014). The questions formulated to identify disability in adults have been finalised and used in several countries, including in the South African Census (Madans *et al*. 2011; Madans & Loeb 2013).

The current focus of the WG/UNICEF module is on developing a measurement instrument for children based on the ICF, which is presently in the piloting phase in various countries (Guzman & Salazar 2014). The most current, pre-final version (called the 2013 WG/UNICEF module on child functioning) used in this study is a screening instrument that can be used for children between 2 and 17 years, which is a parent-reported measure developed for population-based surveillance, and it consists of seven domains, namely seeing, hearing, communication/comprehension, walking, learning, behaviour and playing (WG/UNICEF 2013). To date, limited research has been carried out with regard to sensitivity and specificity of the 2013 WG/UNICEF module (Madans *et al*. 2011; Madans & Loeb 2013), especially in a South African setting (Statistics South Africa 2013).

## Aim of the study

Because there is no measurement instrument standardised for the South African population to address this need, the United Nations Development Programme South Africa, in conjunction with the Department of Women, Children and People with Disability (DWCPD), released a procurement notice. It advises Stats SA to develop a module to measure disability among children aged 0–4 years, for future inclusion in the General Household Surveys and the National Census (Statistics South Africa 2014b). In response to notice, Stats SA has remodelled their work to align it with WG/UNICEF module framework and their module under development (Statistics South Africa 2014b reference).

The ‘gap’ identified in research is that there are no comparable, international or national, population-based childhood disability measures that are methodologically sound and compatible with the ICF (Elwan 1999; Madans *et al*. 2011). This situation consequently leads to a paucity in epidemiological information, compromising the effective healthcare management of all children (Madans *et al*. 2011).

The question arose as to whether the WG/UNICEF module is sensitive and specific enough as a population-based surveillance instrument to identify children below 5 years of age living with disabilities in a rural context of a developing country such as South Africa. As a result, aligned with international work conducted by the WG and national initiatives of Stats SA in the field of population-based surveillance, the authors aimed to report on a pilot study that investigated the sensitivity and specificity of translated versions of the ASQ-III and the WG/UNICEF module as parent-reported measures, to identify early childhood disabilities in children 24–48 months, in a rural area of South Africa (Free State, Xhariep District) to determine their appropriateness for population-based studies in similar contexts.

## Methods

The study used a descriptive design. Ethical clearance was obtained from the Ethics Committee of the Faculty of Health Sciences at the University of the Free State (ECUFS stud. no. 14/2014), and permission was given by the Xhariep District Municipal Offices in Trompsburg, the Regional Offices of SASSA in Bloemfontein, the respective publishers and developers of the measurement instruments and consent was obtained from the participants.

The study was implemented in six towns in the Kopanong Municipal area situated within the Xhariep District. This setting is defined as rural, and here large socioeconomic inequalities prevail; a large majority of the population is unemployed, has low socioeconomic status, is poorly educated and relies almost exclusively on the poorly resourced public health services (David, Tavasci & Marais 2006; Lehohla 2002). This area is therefore representative of many others in South Africa and other developing countries. The population for this study was identified as follows: (1) a heterogeneous group of primary carers (residing in the same household as the child); (2) of children, aged 24–48 months; (3) from the Xhariep District; (4) proficient in Afrikaans, English or Sesotho; and (5) receiving one of the social grants paid by SASSA. These grants include (1) the child support grant (CSG) introduced in 1998 to help alleviate income-poverty experienced by many children between birth and 18 years in South Africa (Dlamini, Ntuli & Petersen 2012–2013); (2) a foster care grant (FCG) for children ordered by the court to remain in the care of foster parents (Hall & Proudlock 2010); or (3) the care dependency grant (CDG) for children between the age of 1 and 18 years who have mild/severe disabilities and require permanent care from a carer (Dlamini *et al*. 2012–2013).

The type of social grant received by the primary carers (in this study specifically a CDG or not) was considered to be the gold standard for this study. A more direct (hands-on), expert clinical assessment of each child would have been a more ideal approach, which, because of financial and time constraints, was not possible. The CDG, however, is based on a comprehensive medical examination to confirm the child’s disability status, and therefore this was deemed appropriate for the purpose of this study. Furthermore, the data regarding the SASSA recipients were easily accessible, without charge and not reliant on clinicians. It is important to note that although the FCG assumes a child to be typically developed, it does not exclude children with mild disabilities, which are often not as apparent and therefore not detected until later years (Mathews *et al*. 2014).

Non-probability convenience sampling was used to identify the sample population of 50 primary carers. A list of beneficiaries in the Xhariep District was obtained from the Regional Offices of SASSA in Bloemfontein in 2014. These social grant beneficiaries on their database were primary carers with typically developed children (indicated by a CSG and an FCG) and those with children with a disability (indicated by the CDG) between the ages of 24 and 48 months. Because of an unexpected, small number of children on the initial SASSA database, the study population consisted of all the primary carers registered either on the Regional or the District SASSA system to receive either one (or more) of the three above-mentioned grants. This sample represents this vulnerable population dependent on social security grants in order to support their children. Because of time and financial constraints, only the 24–48 month sections of the ASQ-III and WG/UNICEF module’s questions were translated and accordingly became the age group focussed on in this study.

A community health worker or SASSA representative in each of the identified towns assisted with locating participants. Data collection procedures took place at multipurpose centres, including community halls, local crèches and SASSA branches in the Xhariep District. Although different venues were used, a standard setup controlling external factors was exercised by the researchers to warrant reliability. The researchers were paired into three subgroups where prescribed roles and responsibilities were maintained. Within these subgroups, one researcher conducted the structured interview, while the other recorded the responses on the respective data forms and assisted the child where needed, in order to allow the carer to focus on the interview. Prior to the structured interviews, information was given to the carer regarding this study and informed consent was obtained. The importance of answering all questions truthfully was explained to the participants, and they were ensured that the information would be kept strictly confidential.

Structured interviews based on parent-reported questionnaires in the participants’ preferred language (Sesotho, English or Afrikaans) were conducted to ensure consistency and compensate for illiteracy. The structured interviews varied between 20 and 30 minutes, limiting the effect of fatigue on the carer’s concentration span. The child remained under the supervision of the carer in order to attend to their basic needs (such as diaper change, hunger or thirst). During the structured interviews, the procedures were to obtain demographic information, conduct the WG/UNICEF module, followed by the ASQ-III, and capture responses. In the case where the ASQ-III and/or the WG/UNICEF module detected developmental delays or disability, and the child was not already on a CDG (indicating disability), the carers received feedback and were referred to the appropriate public healthcare services.

For the purpose of this study the demographic questionnaire provided information regarding grant type, preferred language, age and educational level of the carers.

Because of the multilingual context of the study setting, forward–backward translations of the ASQ-III and WG/UNICEF module to Afrikaans and Sesotho were completed to accommodate all participants. The forward–backward translation process aimed to allow the participants to better understand the questions in their preferred language. After three interviews were performed, it was found necessary to adjust the length of the questions, simplify the vocabulary and substitute examples with more culturally relevant concepts than those used in the ASQ-III.

The data analysis was performed by the Department of Biostatistics, University of the Free State. Descriptive statistics, namely frequencies and percentages for categorical data, and medians and percentiles for continuous data, were calculated. Sensitivity, specificity, positive and negative predictive values (PPV and NPV) and likelihood ratios were calculated by comparing the scores obtained on the parent-reported questionnaires to the type of social grant received by the primary carers. These calculated values are described by means of 95% confidence intervals (CIs). Furthermore, the Youden Index, a diagnostic tool, was also used to determine the superior measurement instrument (Hawass 1997). Kappa was calculated to describe the agreement between ASQ-III and WG/UNICEF module, with 95% CI for kappa.

## Results and discussion

The sample consisted of 50 primary carers, of which 5 were recipients of the CDG (indicating a child with disability), while the remaining 45 were recipients of either a CSG or an FCG. The small sample size (reflected by the wide CIs in Table 1) was a limitation of this study and could be attributed to the limited resources and time constraints. However, this was a pilot study forming part of a larger, proposed rural birth-cohort study.

**TABLE 1:** Ages and Stages Questionnaire Third Edition and the Washington Group on Disability Statistics/UNICEF module calculated diagnostic accuracy parameters.

Accuracy parameters	Measurement instruments		95% Confidence intervals	
	
	ASQ-III	WG/UNICEF	ASQ-III	WG/UNICEF
Sensitivity	60.0%	60.0%	[15%; 95%]	[15%; 95%]
Specificity	95.6%	84.4%	[85%; 99%]	[71%; 94%]
Predictive value				
Positive	60.0%	60.0%	[15%; 95%]	[7.0%; 65%]
Negative	95.6%	84.4%	[85%; 99%]	[83%; 99 %]
Likelihood ratio				
Positive	13.5	3.857	[2.92; 62.48]	[1.44; 10.36]
Negative	0.419	0.474	[0.14; 1.23]	[0.16; 1.40]
Youden index	0.5	0.4	-	-

*Source*: Authors’ own work

ASQ-III, Ages and stages questionnaire third edition; WG/UNICEF, Washington Group on Disability Statistics/UNICEF.

The carers’ ages ranged from 20 to 65 years, with a median age of 29.5 years. The relationship of the child to the carer comprised 36 mothers, 13 grandparents/other relatives and 1 foster parent. Most of the children referred to in the questionnaires were male (62%).

Regarding the educational level of this population (Table 2), the majority (*n* = 38; 76%) had some level of secondary school education. Approximately one-third of the carers (*n* = 17; 34%) had obtained the Grade 12 National Senior Certificate, opposed to only four (8%) who had no formal education at all.

**TABLE 2:** Educational level of the carers (*n* = 50) of children with disabilities.

Level of education	*n*	%
None	4	8
Primary school (Grade 4–7)	8	16
Secondary school		
Grade 8–9	12	24
Grade 10–11	9	18
Grade 12	17	34

*Source*: Authors’ own work

Despite the fact that the carers were predominantly Sesotho-speaking, the majority preferred to participate in the study in either Afrikaans (*n* = 31; 62%) or English (*n* = 18; 36%). Only one interview was conducted in Sesotho through the use of a translator. It was evident, however, during the present study that the WG/UNICEF module’s questions were easily understood, as compared with the questions and terminology of the ASQ-III that were not always clearly understood. Therefore, the ASQ-III questions were reduced to the core concept and asked in a more direct manner. Furthermore, predetermined demonstrations and culturally appropriate examples were consistently provided in order to facilitate better understanding of concepts. For example, threading macaroni onto a string (when macaroni is uncommonly used for anything other than consumption among these participants) was consistently replaced by threading beads onto a string, which was demonstrated. According to the developers of the ASQ-III, the language used to formulate the questions had been based on a Grade 6 level in the USA (Squires *et al*. 2009), as compared with the educational level of the participants of whom 76% had completed at least Grade 7. Thus, it may be of value for qualitative research to further develop these questions into conceptual equivalent versions for the South African population.

With regard to the specific question ‘ease of questionnaire’, the majority of the carers in our study found the ASQ-III (89.8%) and WG/UNICEF module (94%) easy to answer and complete, which is similar to the results in other studies (Rydz *et al*. 2006). The reason for the few carers in our study that struggled to understand these questions could be because of the complexity of ‘not knowing how to respond’, or based on a different understanding and interpretation of terms, often found when conceptual equivalence of terms has not been established for a specific population (Schneider 2012). Perhaps in this study the concept and wording of the specific question ‘ease of questionnaire’ were misinterpreted or misunderstood and instead testified as their ‘knowledge of the child’ (Rydz *et al*. 2006), which emphasises the importance of further research.

According to Altman and Bland (1994), sensitivity is ‘the proportion of true positives that are correctly identified by the test’, while specificity is defined as ‘the proportion of true negatives that are correctly identified by the test’. For a good test, both sensitivity and specificity must be high when compared with the gold standard. These values showed that both the measurement instruments are specific, although not as sensitive.

A positive predictive value (PPV) is the proportion of children who are identified by the measurement instruments as having a disability and who are, therefore, correctly classified as CDG recipients. A NPV is the proportion of children who do not have a disability (typically developed) and who received either a CSG or an FCG (Altman & Bland 1994). The PPVs of the ASQ-III and the WG/UNICEF module were 60.0%, while the NPV of the ASQ-III was 95.6% and that of the WG/UNICEF module was 84.4%.

The ASQ-III has been designed as a more clinical measure, used to measure developmental milestones of typically developed children and to identify developmental delays (Gollenberg *et al*. 2009; Limbos & Joyce 2011; Squires *et al*. 2009). The present study has revealed its potential use also for identifying childhood disabilities, given its clinical useful score of 60% for sensitivity (identifying children with disability) and a high score of 95.6% for specificity (identifying typically developed children). This study has therefore confirmed the additional use of the ASQ-III scantily reported in the literature and the value of further exploring it in future (Tsai *et al*. 2006). The ASQ-III was the better test, with the highest Youden Index Value (Hawass 1997).

The WG’s current focus is on finalising the WG/UNICEF module as a measurement instrument for children based on the ICF (WG/UNICEF 2014). The WG/UNICEF module has been cognitively tested in Mumbai (India), Montenegro, Belize, Oman and the USA. Field testing is ongoing and data are currently being analysed from field tests in Mexico, El Salvador, Samoa and Serbia (WG/UNICEF 2014). The present study reports on an initial attempt to contribute to the work of the WG on an international level. Evidence of this study shows a sensitivity of 60% and specificity of 84.4%, and although no literature is yet available to verify whether or not these results are in line with other WG/UNICEF module studies, it agrees moderately (ASQ-III vs WG/UNICEF module: Kappa: 0.62 [0.32; 0.91]) with the ASQ-III measure, which indicates a potentially high level of usefulness for large-scale, population-based surveillance purposes (Schneider *et al*. 2009; WG/UNICEF 2014). It also holds promise as a preliminary screening tool for smaller-scale, community-specific surveillance investigations.

The WG/UNICEF module does not require the same professional skills and question clarification to administer as the ASQ-III but yields results close to a clinical instrument. The advantages of the WG/UNICEF module are that it can be administered by non-clinical staff, and a particular feature is that in our study, it revealed promising results even for a rural, low socioeconomic, relatively limited educated population (WG/UNICEF 2014). This feature is of particular importance for public health surveillance in South Africa, because it is precisely those populations who are fully reliant on state-funded medical services.

Our results regarding parent-reported measures have reflected the value of involving the carers during early childhood developmental (ECD) screening for future clinical, community-specific and population-based surveillance (Rydz *et al*. 2006). The ‘parent-reported’ information provided results without the need of a ‘hands-on’ clinical professional, yielded quick results and gave an accurate perspective regarding the child and was not dependent on the cooperation of the child on that day.

Although both the instruments are parent-reported and appropriate for children under 5 years of age, the WG/UNICEF module (the more population-based measure) as compared with the ASQ (more clinically oriented measure) has the same sensitivity (60%) and approximately the same specificity, indicating moderate agreement. Because the WG/UNICEF module is quicker to administer, easier to understand and based on the ICF, although becoming available in its final format (WG 2014) only recently, it can be considered as an appropriate population-based measure for similar contexts in South Africa and internationally.

Our results verified that both the measurement instruments were specific in terms of identifying children without disabilities, although not as sensitive in identifying children with disabilities. The implication is that children without disabilities are confirmed as such and those with disabilities are not as consistently detected. This lower sensitivity rate should therefore be considered in future population-based studies, and possible accommodations should be considered, such as making use of more than one parent-reported instruments and including/verifying the parent information with the social grant information - if available.

## Conclusion

This pilot study is the first of its kind conducted in South Africa to address the gap in parent-reported measures in the field of population-based surveys to identify disabilities in children under 5 years of age.

Our findings provide useful information for Stats SA for the development of questions Stats SA intends to include, aligned with the WG/UNICEF module, in its next census in 2021, which will enable Stats SA to capture the extent of disabilities of children under 5 years of age, information that has not been reported in the official figures from the 2011 census. Not only will it help to inform the national institutions responsible for providing support to these children, but in addition, it could provide Stats SA with internationally comparable statistics on childhood disability.

It is recommended, however, that future research and development continue with the WG/UNICEF module to enhance its conceptual equivalence and sensitivity for future larger-scale, population-based studies in South Africa and internationally. By the development of sound measures, internationally comparable and compatible with the ICF, we can obtain reliable statistical epidemiological information leading to better management and provision of services to children under 5 years of age living with disabilities, and giving every child the opportunity to achieve his/her optimal potential.
